# Measurement of tumor volume is not superior to diameter for prediction of lymph node metastasis in early gastric cancer with minute submucosal invasion

**DOI:** 10.18632/oncotarget.22894

**Published:** 2017-12-04

**Authors:** Jeung Hui Pyo, Sun-Ju Byeon, Hyuk Lee, Yang Won Min, Byung-Hoon Min, Jun Haeng Lee, Kyoung-Mee Kim, Hyeon Seon Ahn, Kyunga Kim, Yoon-Ho Choi, Jae J. Kim

**Affiliations:** ^1^ Center for Health Promotion, Samsung Medical Center, Seoul, Korea; ^2^ Department of Pathology and Translational Genomics, Samsung Medical Center, Sungkyunkwan University School of Medicine, Seoul, Korea; ^3^ Department of Medicine, Samsung Medical Center, Sungkyunkwan University School of Medicine, Seoul, Korea; ^4^ Statistics and Data Center, Research Institute for Future Medicine, Samsung Medical Center, Seoul, Korea

**Keywords:** tumor diameter, tumor volume, lymph node metastasis, early gastric cancer, endoscopic resection

## Abstract

**Background/Aim:**

The current indication for endoscopic resection in early gastric cancer (EGC) with minute (< 500 µm) submucosal invasion is based on tumor diameter, which may be insufficient to predict lymph node metastasis (LNM). We investigated whether tumor volume might more accurately predict LNM in EGC with minute submucosal invasion.

**Materials and Methods:**

Among patients who underwent gastrectomy for gastric cancer, 346 with well/moderately differentiated EGC with submucosal invasion <500 µm were evaluated. Three-dimensional tumor volume was calculated using an endoscopically resected specimen and compared with 1-dimensional tumor diameter. Predictive ability of tumor diameter or volume for LNM was evaluated using receiver operating characteristic curve analysis.

**Results:**

Tumor diameter and volume predicted LNM with an area under the curve (AUC) of 0.567 and 0.589, respectively. AUC, sensitivity, specificity, positive and negative predictive values, and accuracy of the 2 models were not significantly different. Tumor diameter ≥ 3 cm showed a significant association with LNM (odds ratio [OR], 2.57; 95% confidence interval [CI], 1.01–6.57; *P* = 0.049), whereas a tumor volume cutoff value of 752.8 cm3 showed no significant association with LNM (OR, 1.52; 95% CI, 0.59–3.88; *P* = 0.385).

**Conclusions:**

Tumor volume had no advantage over diameter for predicting LNM in well/moderately differentiated EGC with minute submucosal invasion.

## INTRODUCTION

Endoscopic resection is considered to be the optimal treatment in select patients with early gastric cancer (EGC) [1, 2]. The criteria for patient selection are based on the Japanese treatment guideline [3]. However, the limited ability to predict lymph node metastasis (LNM) in patients undergoing endoscopic resection for EGC remains a major obstacle, and more accurate prediction plays an important role in determining whether the patient should undergo endoscopic resection or surgery. Patients with gastric cancer with submucosal invasion have considerably high incidence of LNM (10%–20%) [4, 5], and as such, have been excluded from endoscopic resection. However, Gotoda [4] proposed an expanded indication for endoscopic submucosal dissection (ESD) including minute submucosal cancer (pT1b, SM1, < 500 µm from the muscularis mucosae) with tumor size ≤ 3 cm and differentiated histology [3]. However, recent studies have reported high incidence of LNM in tumors satisfying these criteria, and thus, the validity of the expanded indication remains controversial [6, 7].

Among the dimensional variables of the primary tumor, maximum tumor diameter and invasion depth are considered to be important predictors for LNM and have been included in endoscopic resection criteria. However, this approach lacks the capacity for panoramic investigation. Recent studies regarding tumors at sites other than the stomach have suggested that tumor diameter may not precisely reflect tumor burden and that 3-dimensional tumor volume may better predict LNM [8–11]. In particular, histologic or radiologic tumor volume in nasopharyngeal, prostate, gastric, and gynecologic cancers has been reported to be associated with prognosis [10, 12–17].

Therefore, the aim of this study was to investigate whether 3-dimensional tumor volume might predict LNM more accurately than 1-dimensional tumor diameter in well/moderately differentiated EGC with minute submucosal invasion.

## RESULTS

### Baseline characteristics according to LNM

From patients who underwent gastrectomy for gastric cancer (*n* = 8352), we excluded those with mucosal cancer (*n* = 4735), submucosal cancer with invasion depth ≥ 500 µm (*n* = 3241), and undifferentiated histology (including poorly differentiated adenocarcinoma and signet ring cell carcinoma; *n* = 30). A total of 346 patients with well/moderately differentiated EGC with SM1 depth of invasion were included in this study, of whom 19 (5.5%) had LNM. Mean patient age was 61.0 (SD, 9.4) years, 281 patients (81.2%) were men, and 65 (18.8%) were women. Comparison of baseline characteristics between patients with or without LNM is shown in Table 1. There were no significant differences in age, sex, or extent or approach of surgery between patients with or without LNM. Tumor location, macroscopic size, and intestinal or mixed type of Lauren’s classification also showed no significant differences. Tumors with LNM were larger in diameter (3.2 ± 1.5 cm vs 2.9 ± 1.6 cm), deeper in depth (286.7 ± 110.1 µm vs 262.4 ± 132.1 µm), and larger in volume (974.6 ± 852.0 cm^3^ vs 845.2 ± 1154.1 cm^3^) compared with tumors without LNM, but the differences were not significant. The proportion of patients with lymphovascular invasion, however, was significantly higher in those with LNM (47.4% vs 11.6%, *P* < 0.001).

**Table 1:** Baseline characteristics of patients

	LNM (−) (*n =* 327)	LNM (+) (*n =* 19)	*p*-value
Age (years), mean ± SD	61.2 ± 9.3	58.7 ± 10.9	0.120
Sex, *n* (%)			
Male	266 (81.4)	15 (79.0)	0.765
Female	61 (18.7)	4 (21.1)	
Multiple gastric cancer, *n* (%)			
No	303 (92.7)	19 (100.0)	0.382
Yes	24 (7.4)	0 (0)	
Extent of surgery, *n* (%)			
Distal gastrectomy	278 (85.0)	17 (89.5)	1.000
Proximal gastrectomy	5 (1.5)	0 (0)	
Total gastrectomy	44 (13.5)	2 (10.5)	
Surgical approach			
Open	301 (92.1)	18 (100.0)	1.000
Laparoscopic	26 (8.0)	0 (0)	
Number of dissected lymph nodes, mean ± SD	36.3 ± 12.6	31.8 ± 9.9	0.110
Tumor location			
Upper third	31 (9.5)	3 (15.8)	0.608
Middle third	95 (29.1)	6 (31.9)	
Lower third	201 (61.5)	10 (52.6)	
Macroscopic type, *n* (%)			
Elevated	39 (11.9)	5 (21.1)	
Flat	22 (6.7)	2 (10.5)	0.457
Depressed	233 (71.2)	15 (63.2)	
Mixed	33 (10.1)	1 (5.3)	
Tumor diameter (cm), mean ± SD	2.9 ± 1.6	3.2 ± 1.5	0.327
Tumor width (cm), mean ± SD	1.9 ± 1.2	1.9 ± 0.9	0.581
Depth of invasion (μm), mean ± SD	262.4 ± 132.1	286.7 ± 110.1	0.354
Tumor volume (cm^3^), mean ± SD	845.2 ± 1154.1	974.6 ± 852.0	0.194
Lauren’s classification, *n* (%)			
Intestinal	325 (99.4)	18 (94.7)	0.156
Mixed	38 (0.6)	1 (5.3)	
Lymphovascular invasion, *n* (%)			
Negative	289 (88.4)	10 (52.6)	< 0.001
Positive	42 (11.6)	9 (47.4)	

*LNM*, lymph node metastasis; *SD*, standard deviation.

### Tumor dimensional variables for predicting LNM

LNM rate according to tumor diameter or volume is shown in Table 2. There was no LNM for tumors ≤ 0.5 cm in diameter or ≤ 50 cm^3^ in volume. Maximum tumor diameter and volume without LNM was ≤ 0.9 cm and ≤ 65.5 cm^3^, respectively. LNM rate of tumors satisfying the expanded endoscopic resection criteria (well/moderately differentiated, SM1, ≤ 3 cm) was 3.6% (8/221).

**Table 2:** Lymph node metastasis rate according to the tumor length and volume

Tumor diameter (cm)	Each group (*n*, %)	Accumulation (*n*, %)	Tumor volume (cm 3)	Each group (*n*, %)	Accumulation (*n*, %)
≤ 0.5	0/4 (0)	0/4 (0)	≤ 50	0/21 (0)	0/21 (0)
0.5–1.0	1/20 (5.0)	1/24 (4.2)	50–100	2/29 (6.9)	2/50 (4.0)
1.0–1.5	3/27 (14.8)	4/51 (7.8)	100–200	2/49 (4.1)	4/99 (4.0)
1.5–2.0	2/67 (14.9)	6/118 (5.1)	200–400	1/76 (1.3)	5/175 (2.9)
2.0–2.5	1/53 (1.9)	7/171 (4.1)	400–800	6/84 (7.1)	11/259 (4.2)
2.5–3.0	1/50 (2.0)	8/221 (3.6)	800–1600	4/49 (13.3)	15/308 (4.9)
3.0–3.5	1/26 (3.8)	9/247 (3.6)	1600–3200	4/30 (12.1)	19/338 (5.6)
3.5–4.0	6/31 (19.4)	15/278 (5.4)	3200–6400	0/7 (0)	19/345 (5.5)
> 4.0	4/68 (5.9)	19/346 (5.5)	> 6400	0/1 (0)	19/346 (5.5)

**Figure 1:**
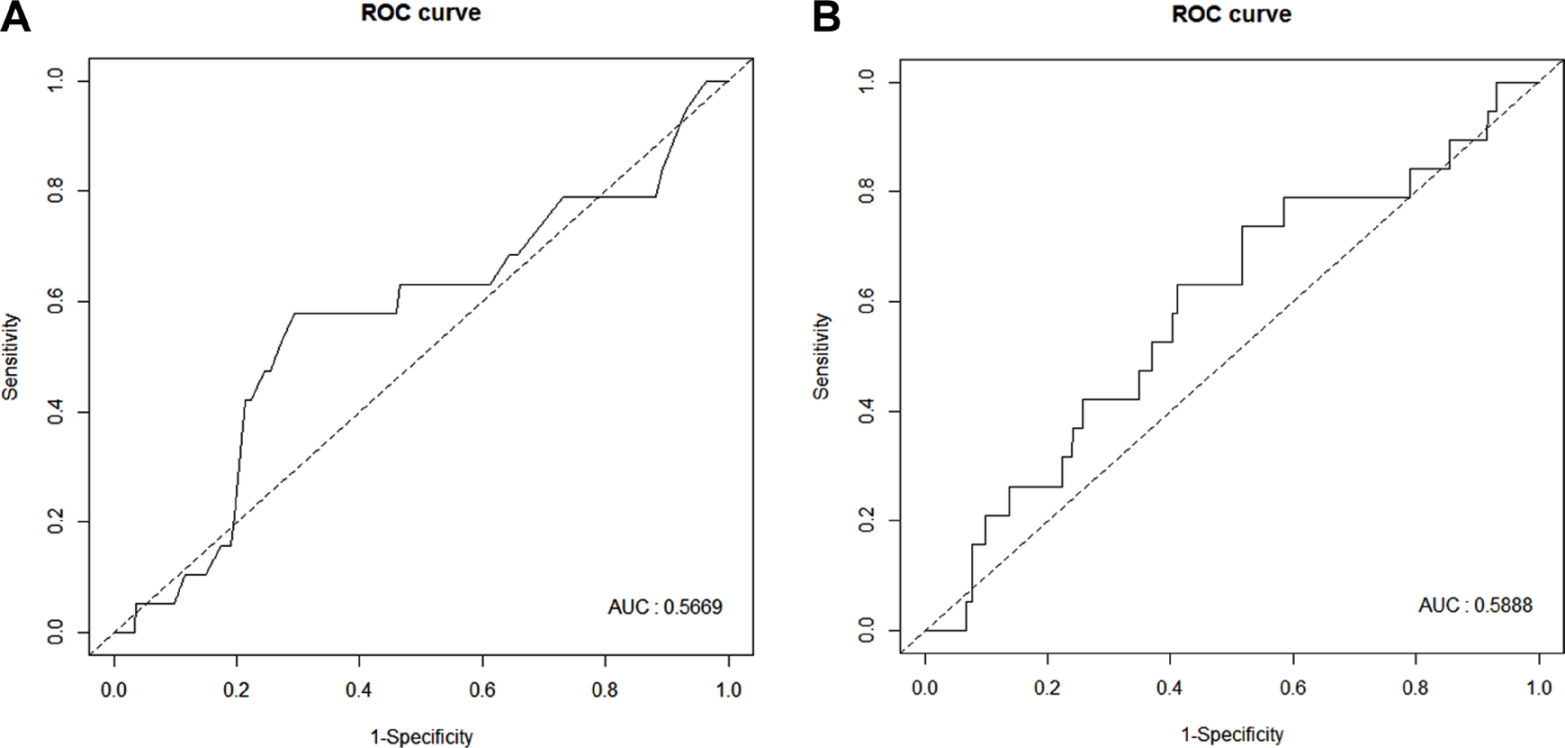
Receiver operating characteristic curve analysis of tumor diameter (**A**) and volume (**B**).

Associations between 1-dimensional tumor diameter and 3-dimensional tumor volume as continuous variables for LNM were tested using logistic regression analysis. Neither diameter (odds ratio (OR), 1.01; 95% confidence interval [CI], 0.84–1.44; *P* = 0.485) nor volume (OR, 1.00; 95% CI, 1.00–1.00; *P* = 0.631) showed a significant association with LNM. Ability to predict LNM was evaluated using receiver operating characteristic (ROC) curve analysis (Figure 1). Tumor diameter and volume predicted LNM with an area under the curve (AUC) of 0.567 and 0.589, respectively. Comparison of the AUCs of the 2 models (diameter vs volume) using the Delong’s test showed no significant difference (*P* = 0.601). Moreover, tumor diameter and volume showed no significant differences in sensitivity, specificity, positive and negative predictive values, or accuracy (Table 3).

**Table 3:** Sensitivity, specificity, positive predictive value, negative predictive value, and accuracy for the cutoffs of the 1D and 3D measurement

Cutoff	3 cm of 1D	752.8 cm 3 of 3D	*p*-value	Method
Sensitivity (%)	58	42	0.180	McNemar’s test
Specificity (%)	65	68	0.310	McNemar’s test
Positive predictive value (%)	9	7	0.318	Bennett’s test
Negative predictive value (%)	96	95	0.239	Bennett’s test
Accuracy (%)	65	66	0.371	McNemar’s test

*1D*, one-dimensional; *2D*, two-dimensional.

The optimal cutoff value of tumor volume, determined using the Youden index and validated using 10,000 bootstrap analyses, was 752.8 cm^3^ (95% CI, 742.8–762.7). Using the 1-dimensional tumor diameter cutoff value of the expanded ESD criteria (3 cm) and the 3-dimensional tumor volume cutoff value from our study (752.8 cm^3^), patients were grouped and the associations with LNM were analyzed using logistic regression analysis (Table 4). Tumor diameter ≥ 3 cm showed a significant association with LNM (OR, 2.57; 95% CI, 1.01–6.57; *P* = 0.049), whereas tumor volume ≥ 752.8 cm^3^ showed no significant association with LNM (OR, 1.52; 95% CI, 0.59–3.88; *P* = 0.385).

**Table 4:** Risk factors for lymph node metastasis in minute submucosal cancer

	Odd ratio	95% CI	*p*-value
Age (years), mean ± SD	0.96	0.92–1.01	0.119
Sex, *n* (%)			
Male	1.00		0.795
Female	0.86	0.28–2.68	
Multiple gastric cancer, *n* (%)			
No	1.00		0.973
Yes	< 0.01	< 0.01– >999.9	
Tumor location			
Upper third	1.00		
Middle third	0.71	0.17–3.00	0.644
Lower third	0.78	0.21–2.84	0.704
Macroscopic type, *n* (%)			
Elevated	1.00		
Flat	0.89	0.15–5.24	0.894
Depressed	0.50	0.15–1.64	0.253
Mixed	0.30	0.03–2.78	0.286
Tumor diameter (cm), mean ± SD	1.10	0.84–1.44	0.485
Tumor diameter group			
≤ 3 cm	1.00		0.049
> 3 cm	2.57	1.01–6.57	
Tumor width (cm), mean ± SD	0.99	0.66–1.48	0.943
Depth of invasion (μm), mean ± SD	1.00	1.00–1.00	0.432
Tumor volume (cm 3), mean ± SD	1.00	1.00–1.00	0.631
Tumor volume group			
≤ 752.8 cm^3^	1.00		0.385
> 752.8 cm^3^	1.52	0.59–3.88	
Lauren’s classification, *n* (%)			
Intestinal	1.00		0.078
Mixed	9.03	0.78–104.30	
Lymphovascular invasion, *n* (%)			
Negative	1.00		< 0.001
Positive	6.85	2.62–17.91	

*SD*, standard deviation; *CI*, confidence interval.

## DISCUSSION

This study evaluated the predictive value of tumor volume for LNM, compared with the conventionally used tumor diameter, using data of gastrectomy with well/moderately differentiated SM1 (< 500 µm) gastric cancer. We hypothesized that tumor volume would help clinicians to predict LNM more accurately than tumor diameter when choosing the optimal treatment modality for individual cases after endoscopic resection. However, tumor volume failed to predict LNM, and the performance of the 2 models showed no significant differences.

For intramucosal EGC, endoscopic resection is the current standard of treatment. For EGC with submucosal invasion meeting the expanded indication, however, oncologic clearance of ESD remains controversial [18–20]. Previous studies have suggested several different methods to identify those with very low risk of LNM among patients with EGC with minute submucosal invasion. Eom et al. [21] reported that a cutoff value of 300 µm had the highest predictive value (98%) and suggested a range reduction of the currently used criterion of 500 µm for depth of submucosal invasion. Kim et al. [22] showed that 2-dimensional tumor size predicted LNM better than the currently used 1-dimensional size in well/moderately differentiated EGC with minute submucosal invasion (< 500 µm).

Solid tumors are 3-dimensional structures with unequal rates of tumor spread in different directions and planes, and as such, diameter does not accurately reflect total tumor volume or total malignant cell burden [8]. Therefore, several studies have been conducted to assess the ability of tumor volume to predict LNM [8, 10, 11, 14, 23]. A recent study showed that gross tumor volume measured on multidetector computed tomography (CT) was associated with regional LNM and N categories in resectable gastric adenocarcinoma [24]. EGC is frequently undetectable on CT, and planar size estimation by endoscopic examination is the only way to determine tumor extent. However, after endoscopic resection, the resected specimen undergoes pathologic evaluation, and patients with noncurative resection are recommended to undergo additional surgery for risk of LNM. If tumor volume measured using endoscopically resected specimens predicted LNM better than 1-dimensional size, it could be a useful tool for determining treatment strategy after endoscopic resection. This was, to our knowledge, the first study to evaluate the association between tumor volume and LNM, and to compare the predictive ability for LNM of 1- or 3-dimensional size, in EGC with minute submucosal invasion. Three-dimensional volume of the primary tumor was calculated using endoscopically resected specimens, with precise depth of tumor invasion assessed by expert pathologists. However, this study showed that sensitivity, specificity, predictive value, and accuracy were not significantly different between 1- and 3-dimensional methods. Our data also confirm the cutoff criterion (tumor size < 3 cm) defined by Gotoda [4].

This study has several limitations. First, the total number of cases was small, including only those with minute submucosal gastric cancer at a single center. Second, the same formula was used in every case to estimate tumor volume. However, tumors have various shapes, such as rectangular, cuboidal, or ellipsoid, and ideally, different formulas should be used to calculate tumor volume [25]. However, this was not considered as this was a retrospective study.

In conclusion, our findings showed that tumor volume had no advantage over tumor diameter for predicting LNM in well/moderately differentiated EGC with minute submucosal invasion, unlike advanced gastric cancer. Future studies with more accurate methods to measure tumor volume may show different results.

## MATERIALS AND METHODS

### Subjects

We used prospectively collected data of patients who underwent gastrectomy for gastric cancer at Samsung Medical Center, Seoul, Korea, from January 2002 to December 2013. Patients underwent open or laparoscopic, subtotal or total gastrectomy depending on tumor location, with D1+ or D2 lymphadenectomy according to the Japanese treatment guideline [3]. Of these patients (*n* = 8352), those with well/moderately differentiated EGC with SM1 depth of invasion were included in this study. This study was approved by the Institutional Review Board of Samsung Medical Center.

### Data collection

Patient data including age, sex, presence of synchronous tumor, extent of surgery (distal, proximal, or total), and surgical approach (open vs laparoscopic) were collected. Number of dissected lymph nodes was described as mean (SD). Tumor location was categorized as upper-, middle-, or lower-third of the stomach. Macroscopic type was reported based on pathologic findings according to the Japanese guideline [18] and categorized into 1 of 4 groups: elevated (I, IIa, I + IIa, IIa + IIb), flat (IIb), depressed (IIc, IIc + III), or mixed (others). Histologic type was classified according to the 2010 World Health Organization classification [26] and categorized according to the Japanese guideline [27]. Differentiated type included papillary adenocarcinoma as well as well- and moderately differentiated tubular adenocarcinoma, while undifferentiated type included poorly differentiated tubular adenocarcinoma and signet ring cell carcinoma. Tumors composed of both differentiated and undifferentiated types were classified according to the quantitatively predominant type [27]. Lymphovascular invasion was defined when tumor emboli were found within a space that was clearly lined by endothelial cells. Lymph nodes larger than 5 mm were cut into 2 pieces and the cut surfaces were examined to determine presence of metastasis in each node. Tumor staging was carried out according to the American Joint Committee on Cancer classification system (7th edition) [28].

### Evaluation of dimensional variables

**Figure 2:**
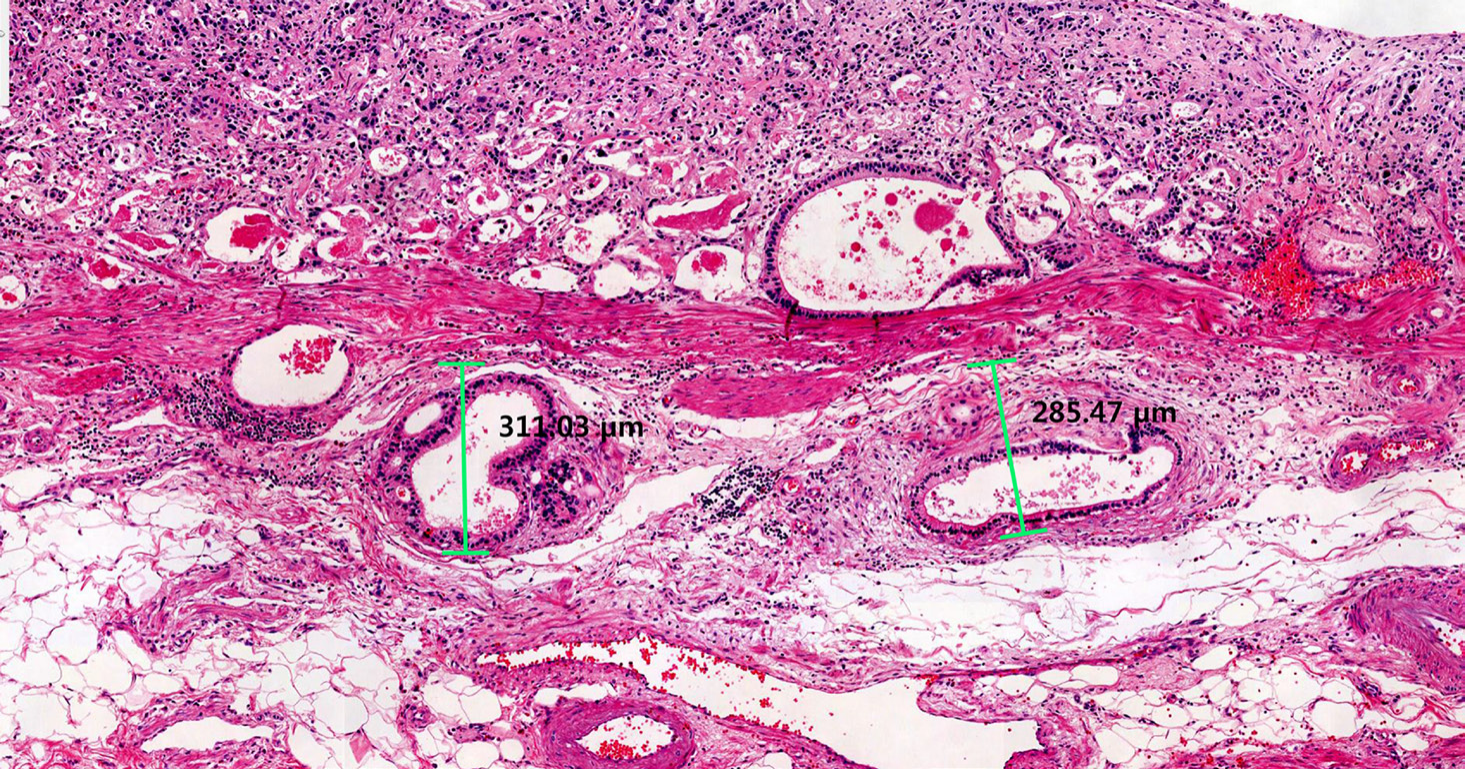
Measurement of depth of tumor invasion Depth of tumor invasion was defined as the depth perpendicular to an imaginary line drawn from the adjacent muscularis mucosae.

Surgical specimens were fixed in 10% formalin, processed, and embedded in paraffin, and then stained with hematoxylin and eosin using the standard protocol. Tumor diameter (or size) was defined as the longest diameter, and tumor width as the maximum width perpendicular to the diameter. Slides with the deepest infiltrated tumor cells were selected and scanned using the Ventana iScan HT slide scanner (Ventana Medical Systems, Tucson, AZ, USA) with a 20× objective lens. Depth of invasion was defined as the depth perpendicular to an imaginary line drawn from the adjacent muscularis mucosae (Figure 2) [29]. Estimated tumor volume was calculated using the following equation: 0.5 × diameter × width × depth [9, 10].

### Statistical analysis

Baseline characteristics were analyzed between patients with or without LNM using the Mann-Whitney *U* test for continuous variables and the Chi-square test or Fisher’s exact test for categorical variables. ROC curve analysis was used to estimate the optimal cutoff value of tumor volume for predicting LNM. The result of ROC analysis was validated by performing the 10,000 bootstrap resampling method. Predictive ability of tumor diameter or volume for LNM was evaluated based on the AUC of the ROC curve, and sensitivity, specificity, positive predictive value, negative predictive value, and accuracy were calculated. DeLong’s test was used to compare the AUCs of the 2 correlated ROC curves [30]. Logistic regression analysis was used to analyze risk factors for LNM. All statistical analyses were performed using SAS version 9.4 (SAS Institute, Cary, NC, USA), and *P* values of < 0.05 were considered statistically significant.

## Abbreviations

CI: confidence interval
CT: computed tomography
EGC: early gastric cancer
ESD: endoscopic submucosal dissection
LNM: lymph node metastasis
OR: odds ratio
